# RALF-FER, a master ligand‒receptor pair in plant health

**DOI:** 10.1007/s44297-024-00040-1

**Published:** 2025-02-20

**Authors:** Xing-Yan Chen, Jia Chen, Fan Xu, Xin-Zhong Cai

**Affiliations:** 1 Zhejiang Key Laboratory of Biology and Ecological Regulation of Crop Pathogens and Insects, Institute of Biotechnology, College of Agriculture and Biotechnology, Zhejiang University, 866 Yu Hang Tang Road, Hangzhou, 310058 China; 2 Hunan Key Laboratory of Plant Functional Genomics and Developmental Regulation, Hunan University, Changsha, China; 3 Hainan Institute, Zhejiang University, Sanya, 572025 China

**Keywords:** FERONIA, RALF, Plant immunity, Pathogen, Crop resilience

## Abstract

Pathogens deliver many effector proteins into the plant apoplast, which helps plants evade pattern recognition receptor (PRR)-mediated surveillance by camouflaging or blocking PRR-triggered signaling. Plants must prioritize immunity or growth and development according to the presence or absence of pathogen-derived effectors. Crosstalk exists between PRR immune signaling pathways and growth and development pathways. A typical example is the signaling pathway of the receptor kinase FERONIA (FER), a core element of a global signaling network. FER interacts with its coreceptors and different Rapid Alkalinization Factor (RALF) peptide ligands to function in various growth and developmental processes and respond to pathogens. Studies on the roles of host FERs in different plant species and those of RALFs derived from both hosts and pathogens are beginning to flourish. Here, we focus on recent advances in FER and RALF in plant‒pathogen interactions, with an emphasis on the mechanisms underlying these interactions. We also present a brief outlook to highlight challenges and perspectives for future research on how to utilize the RALF-FER pair or its related signaling elements as targets to improve crop resistance to pathogens.

## Overview

Plants must sense diverse attacking organisms, including viruses, bacteria, fungi, oomycetes, herbivores, and parasitic plants. At the plasma membrane (PM) of plant cells, cell-surface receptors that are either receptor-like kinases (RLKs) or receptor-like proteins (RLPs) sense extracellular danger signals to activate defense responses [1, 2]. To cause disease, pathogens usually need to evade detection by the host and/or to suppress these immune responses. Extracellular danger signals include pathogen- or microbe-associated molecular patterns (PAMPs or MAMPs), microbial effectors, and patterns originating from the host, namely, damage-associated molecular patterns (DAMPs) and phytocytokines. Increasing evidence shows that pathogens can secrete peptides, act as effectors, or induce host-derived peptides, which act as patterns originating from the host, to suppress or evade pattern-triggered immunity (PTI), which is initiated by RLKs or their related pattern recognition receptors (PRRs; [3–5]). Therefore, the question of how particular phytopathogens utilize variants of these peptides to facilitate host infection and/or disease development has attracted interest, in part, because of their potential to guide pathogen control in agriculture.

Rapidly Alkaline Factors (RALFs) are important peptides that function in almost all plant biological processes, including plant growth, development, reproduction, and abiotic and biotic stress responses. RALF was first identified as a growth inhibitor in tobacco [6]. It is a 5-kDa cysteine-rich peptide with the ability to activate mitogen-activated protein kinase (MAPK) and inhibit root growth [6]. RALF1 was found to inhibit growth by regulating proton pump proteins through its receptor kinase, thereby restricting cell expansion [7]. RALFs were named for their ability to quickly alkalize the extracellular domain of plant cells [6]. Recent studies have revealed that many RALF genes or RALF-like genes also exist in fungal and nematoid genomes [5, 8]. Pathogen RALFs act as immune modulators and hijack host PRR pathways to improve pathogenesis [3, 5].

To date, all identified receptors of RALFs are cell surface RLKs. RLKs contain a variable extracellular domain (ECD) responsible for ligand binding, a single transmembrane domain (TMD), and a cytoplasmic kinase domain (CD; [2, 9]). There are at least 610 RLK members in *Arabidopsis thaliana*, accounting for approximately 2.5% of the Arabidopsis coding genes [10]. *Catharanthus roseus* receptor-like kinase 1-like (*Cr*RLK1L) proteins are a subfamily of RLKs consisting of 17 members in Arabidopsis. The first member was named *Cr*RLK1, as it was cloned from *Catharanthus roseus* plants in Madagascar in 1996 [11]. *Cr*RLK1Ls structurally contain two malectin-like domains (MLDs) in their ECD, a TMD and a CD [12]. FERONIA (FER) is the best-characterized member of the *Cr*RLK1L family, which was initially identified from a pollen tube (PT) reception mutant and was found to be involved in regulating PT elongation to ensure normal fertilization in plants [13–15]. FERONIA is the goddess of fertility in Etruscan mythology [13, 15]. Notably, FER is required for full responsiveness to multiple RALF peptides [16] and can be targeted by pathogenic RALFs from fungi [3] and nematodes [5, 17].

Many reviews and perspectives [12, 18–26] have provided a comprehensive overview of the rapidly unfolding field of FER and RALFs in plants. This review introduces the evolution and multiple functions of FERs and RALFs in plant development and the abiotic stress response and highlights the contributions and mechanisms of RALF-FER in plant‒pathogen interactions.

## Evolution, function, and recognition of the host RALF

RALFs play important roles in various life processes of plants, such as cell extension, signal transduction, root growth, PT development, and biotic and abiotic stress responses [27]. These genes have been identified in various species, including 37 members in Arabidopsis, 43 in rice, 18 in soybean, and 34 in maize [16, 28]. In Arabidopsis, the majority of RALF peptides display growth inhibitory properties. Additionally, 7 RALFs containing the predicted subtilisin-like serine protease SITE-1 PROTEASE (S1P)-cleavage site inhibited elf18-induced reactive oxygen species (ROS) bursts, while the majority of noncleaved RALFs promoted this process, indicating the functional complexity and signaling specificity of the signaling of RALF peptides [16].

The mature RALF polypeptide with biological activity is cleaved and released from the C-terminus of a precursor protein, which has a recognition site RxxL/RxLx (where R is arginine, L is leucine, and x is any amino acid) for the S1P. The N-terminus of the typical RALF preproprotein has a signal peptide, which can guide its secretion to the outside of the cell [6, 29]. Blockage of the cleavage process strongly affects the physiological functions of RALFs. For example, the overexpression of RALF23 in Arabidopsis leads to a significantly dwarfed and fascinating phenotype. However, the overexpression of RALF23 with an RRIL to GGIL mutation does not significantly alter the phenotype. Moreover, S1P mutation inhibits PAMP-induced secretion of RALF23 [30–32]. After being cleaved and released, the mature RALF polypeptide contains two key domains—YISY and four conserved cysteines at the C-terminus. The YISY domain is crucial for its binding to receptors, and the four cysteines can form intramolecular disulfide bonds, which play crucial roles in maintaining the three-dimensional conformation and biological activity of RALFs [5, 33–35]. For example, mutation of the first and third cysteines of RALF22 compromises its activity with respect to ROS induction, MAP kinase activation, root growth suppression, and immunity elicitation [36]. However, as a large family of peptides, RALFs contain atypical members, which do not have an S1P recognition site or have a mutated YISY motif and conserved cysteine residues [29]. The diversity of RALF sequences within and between species indicates the complexity of RALF signaling.

Notably, in addition to being signal molecules, RALFs act as structural components of the nascent cell wall because of their electrical properties. Positively charged LRX–RALF complexes interact with negatively charged pectin (homogalacturonan (HG)). In tip-growing cells (PTs and root hairs (RHs)), RALFs form complexes with purified homogalacturonan and LRXs in a charge-dependent manner. LRX8–RALF4–HG (in PT; [37]) and LRX1–RALF22–HG (in RH; [38]) complexes induce homogalacturonan dewatering and compaction and self-assembly of the cell wall microdomain.

The *RALF* gene family evolved rapidly after the separation of eudicot and monocot species approximately 145 million years ago. Tandem duplication has played a dominant role in the expansion of the Arabidopsis and rice RALF gene families [39]. *RALFs* were under purifying selection according to estimations of the substitution rates of these genes [39]. RALFs (795 RALFs from 51 plant species) can be classified into four major clades. Among them, clades I, II, and III contain features that have been identified to be important for RALF activity, including the RRXL cleavage site and the YISY motif required for receptor binding. Members of clade IV are highly diverged and lack these features. They also exhibit distinct expression patterns and physicochemical properties. The expansion of this RALF–related clade in Brassicaceae is responsible for the large number of RALF genes in *Arabidopsis *[29].

All known receptors of RALFs are members of the *Cr*RLK1L family (Table 1). In Arabidopsis, the RALF1-FER pair was the first identified ligand‒receptor pair [7]. Emerging evidence has shown that other RALFs can also act as FER ligands. These include RALF22 [36], RALF23, RALF17, RALF32, and RALF33 [16, 32].

**Table 1:** Overview of the RALF family in Arabidopsis

Peptide name	Gene ID	S1P site	Receptor	Functions
RALF1	AT1G02900	Yes	FER	Negatively regulate cell expansion and root growth [7]; inhibit stomatal opening and induce its closure [40]; cause Na^+^ overaccumulation to enhance salt toxicity [40]; regulate plant carbon/nitrogen responses in a FER-dependent way [41]
RALF2	AT1G23145	No		
RALF3	AT1G23147	No		
RALF4	AT1G28270	Yes	ANXUR1 (ANX1) ANX2 Buddha’s Paper Seal 1 (BUPS1) BUPS2	Control pollen tube growth and maintain pollen tube integrity [42, 43]
RALF5	AT1G35467	No		
RALF6	AT1G60625	No	FER ANJEA (ANJ) HERCULES RECEPTOR KINASE 1 (HERK1)	Induce the formation of FER-ANJ-HERK1 heteromeric receptor complexes to prevent the arrival of multiple pollen tubes to one ovule to ensure reproductive success [44]
RALF7	AT1G60815	No	FER ANJ HERK1	Induce the formation of FER-ANJ-HERK1 heteromeric receptor complexes to prevent the arrival of multiple pollen tubes to one ovule to ensure reproductive success [44]
RALF8	AT1G61563	No		Inhibit root growth and lead to a severely stunted phenotype [45]; reduce growth and probably participate in cell wall modification in response to severe water deficit and nematode stress [45]
RALF9	AT1G61566	No		
RALF10	AT2G19020	No		
RALF11	AT2G19030	No		
RALF12	AT2G19040	No		
RALF13	AT2G19045	No		
RALF14	AT2G20660	Yes		
RALF15	AT2G22055	No		
RALF16	AT2G32835	No	FER ANJ HERK1	Induce the formation of FER-ANJ-HERK1 heteromeric receptor complexes to prevent the arrival of multiple pollen tubes to one ovule to ensure reproductive success [44]
RALF17	AT2G32885	No	FER	Induce ROS production and positively regulate PTI [32]
RALF18	AT2G33130	Yes		
RALF19	AT2G33775	Yes	ANX1 ANX2 BUPS1 BUPS2	Control pollen tube growth and maintain pollen tube integrity [42, 43]
RALF20	AT2G34825	No		
RALF21	AT3G04735	No		
RALF22	AT3G05490	Yes	FER	Regulate plant salt tolerance [46]; positively regulate PTI and resistance to *Sclerotinia. sclerotiorum* [36]
RALF23	AT3G16570	Yes	FER	Impair BL-induced hypocotyl elongation and inhibit plant growth [31, 32]; regulate plant salt tolerance [46]; reduce growth in response to severe water deficit and nematode stress [45]; inhibit FLS2/EFR-BAK1 complex formation to negatively regulate PTI [32]; induce a complex between FER-LLG1 complex formation to regulate immune signaling [35], activate localized immunity in root [47]
RALF24	AT3G23805	No		
RALF25	AT3G25165	No		
RALF26	AT3G25170	No		
RALF27	AT3G29780	Yes		
RALF28	AT4G11510	No		
RALF29	AT4G11653	No		
RALF30	AT4G13075	No		
RALF31	AT4G13950	Yes		
RALF32	AT4G14010	No	FER	Inhibit plant growth [32]
RALF33	AT4G15800	Yes	FER	Inhibit plant growth [32]; reduce growth in response to severe water deficit and nematode stress [45]; Negatively regulate PTI [32]
RALF34	AT5G67070	Yes	ANX1 ANX2 BUPS1 BUPS2 THESEUS1 (THE1)	Induce pollen tube bursting and sperm cell release at nanomolar concentrations [42]; fine-tune the lateral root initiation [48]; reduce growth in response to severe water deficit and nematode stress [45]; negatively regulate PTI [32]
RALF35	AT1G60913	No		
RALF36	AT2G32785	No	FER ANJ HERK1	Induce the formation of FER-ANJ-HERK1 heteromeric receptor complexes to prevent the arrival of multiple pollen tubes to one ovule to ensure reproductive success [44]
RALF37	AT2G32788	No	FER ANJ HERK1	Induce the formation of FER-ANJ-HERK1 heteromeric receptor complexes to prevent the arrival of multiple pollen tubes to one ovule to ensure reproductive success [44]

This table lists the reported members of the Arabidopsis RALF family and briefly outlines their characteristics and functions. The reported composition of the AtRALF family in different publications and the TAIR10 and UniProt databases. The protein names and corresponding accession numbers used here are based on Abarca’s paper [16]

Pathogens can not only induce host RALF production [47] but also secrete RALFs to control local communication and cell proliferation, growth and differentiation in the host [3]. For example, the rhizobacteria *Pseudomonas syringae* pv. *tomato* DC3000 (DC3000) and *P. protegens* strain CHA0 (CHA0) trigger the maturation and expression of the ligand peptide RALF23 in the transition and elongation zones of roots [47]. Biologically active RALF homologs have been identified within numerous fungal phytopathogens, potentially acting in plant‒pathogen interactions [8]. Certain members of Actinobacteria possess putative secreted proteins with an incorporated RALF peptide domain motif. Bacterial RALF domain-containing proteins also contain a domain homologous to the S1 pertussis toxin subunit [8]. Whether these fungal RALFs originated through horizontal gene transfer or coevolution is not yet clear.

## Evolution of FER in plants

In general, PTI is initiated upon recognition of MAMPs or DAMPs by PRRs. PRR recognition is a key battlefront in plant–pathogen interactions. It is employed by plant cells to recognize and inactivate invading pathogens and is targeted by microbes to overcome the plant immune system, thereby successfully colonizing host cells. One of the best-characterized PRR complexes in plants consists of a *scaffold* receptor (FER), *client* receptors (EF-TU RECEPTOR, EFR; FLAGELLIN-SENSING 2, FLS2), and *client* co-receptors (BRASSINOSTEROID INSENSITIVE 1–ASSOCIATED KINASE 1, BAK1; LORELEI-like-GPI-anchored proteins, LLGs). As a key scaffold receptor of PRRs and a typical receptor of RALFs, FER plays an important role in plant‒pathogen interactions and continues evolution in plants. *FER* and its homologs have been characterized in various higher plants, such as rice, strawberry, and apple [13, 49, 50]. Identification and evolutionary analysis of FERs in different plant lineages, including Charophyta and basal land plant lineages from Angiosperms (Monocots, Eudicots, and *Amborella trichopoda*), Gymnosperms, Ferns, Lycophytes, and Bryophytes, will help to further understand their roles in increased plant immunity.

**Fig. 1:**
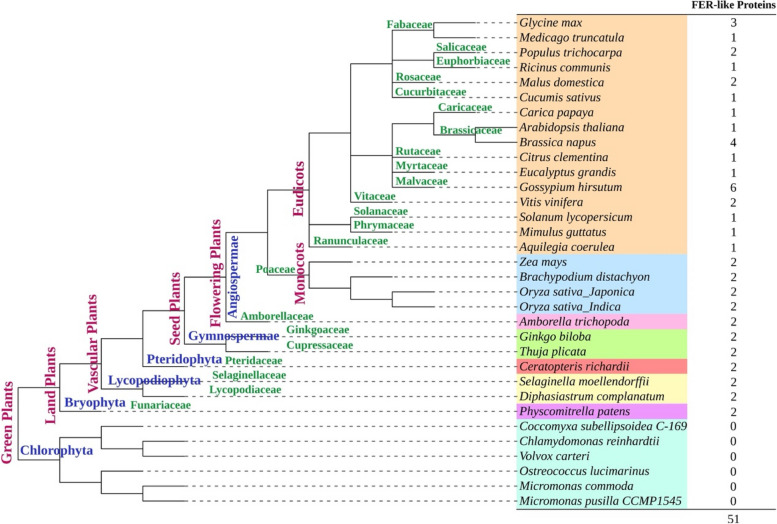
The presence of FER-like sequences within major green lineages. The phylogenetic tree for FER-like proteins of 26 plant species from diverse lineages is shown

Using the complete amino acid sequence of the Arabidopsis FER (AtFER) protein downloaded from the TAIR database as the query sequence, BLASTp was performed in the NCBI or Phytozome database to search for potential homologous protein sequences of AtFER. The *E* value was set to 0.001, and the other parameters were set to default values. After searching, aggregating and de-duplication, 50 FER-like proteins were identified in 26 plant species (including 1 moss, 2 lycophytes, 1 fern, 2 gymnosperms, *A. trichopoda*, 4 monocots and 15 eudicots) in addition to Arabidopsis (Fig. 1). These FER-like proteins have total lengths ranging from 691 to 987 amino acids. Furthermore, we conducted similar BLASTp search analyses in six Chlorophyta genomes and detected no AtFER-like protein that contains the MLD, transmembrane domain, or intracellular kinase domain (Fig. 1). These results indicate that FER might originate from basal vascular plants.

**Fig. 2:**
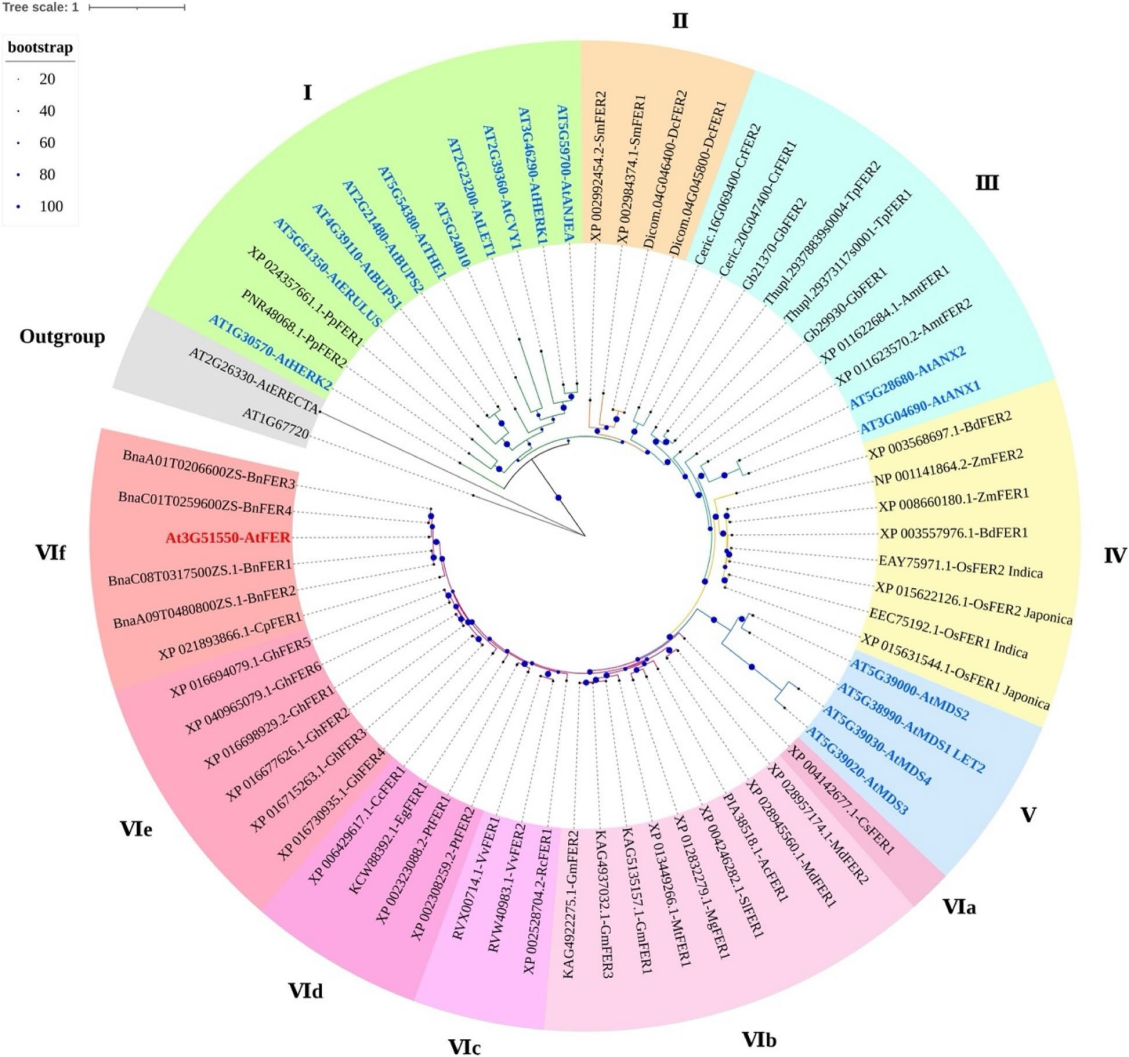
Phylogenetic tree of 67 plant FER-like proteins identified in 26 plant species. The phylogenetic tree was constructed via the maximum likelihood (ML) method with 1000 bootstraps via IQ-TREE software. The tree was divided into seven groups, with group VI divided into six subgroups: a, b, c, d, e, and f. Groups were distinguished by color, with Group I in green, Group II in orange, Group III in blue–green, Group IV in yellow, Group V in blue, and Group VI in red. The subgroups of Group VI were distinguished by color depth, while the outgroup is marked in gray. The blue dots on the evolutionary branch represent the bootstrap values. Abbreviations of the species: At, *Arabidopsis thaliana*; Pp, *Physcomitrella patens*; Mg, *Mimulus guttatus*; Sl, *Solanum lycopersicum*; Ac, *Aquilegia coerulea*; Mt, *Medicago truncatula*; Gm, *Glycine max*; Md, *Malus domestica*; Vv, *Vitis vinifera*; Rc, *Ricinus communis*; Bn, *Brassica napus*; Cp, *Carica papaya*; Gh, *Gossypium hirsutum*; Cc, *Citrus clementina*; Eg, *Eucalyptus grandis*; Pt, *Populus trichocarpa*; Cs, *Cucumis sativus*; Bd, *Brachypodium distachyon*; Os, *Oryza sativa*; Zm, *Zea mays*; Amt, *Amborella trichopoda*; Gb, *Ginkgo biloba*; Tp, *Thuja plicata*; Cr, *Ceratopteris richardii*; Sm, *Selaginella moellendorffii*; Dc, *Diphasiastrum complanatum*

*Cr*RLK1Ls have been identified in a variety of species of angiosperms, gymnosperms, and early diverging lineages [10, 21]. Homologous *Cr*RLK1L sequences have also been identified in the bryophyte *Marchantia polymorpha* and in the charophyte *Chara braunii*, suggesting that *Cr*RLK1Ls appeared before the emergence of land plants [10]. The high morphological complexity of Chara could have resulted from the advent and/or genomic expansion of the *Cr*RLK1L gene family [10]. To analyze the evolution of FER in different plant species, a phylogenetic tree was constructed via the maximum likelihood (ML) method via the full-length sequences of 17 *Cr*RLK1L proteins in Arabidopsis and 50 FER homologous proteins identified in 26 other plant species of representative lineages (Fig. 2). The Arabidopsis LRR-RLK proteins AT1G67720 and RLK ERECTA (AT2G26330) were used as outgroups. The 67 proteins could be classified into six groups (I-VI). Groups I-VI have 12, 4, 10, 8, 4, and 29 members, respectively. Ten non-FER Arabidopsis *Cr*RLK1L members and two FER-like proteins identified in mosses were distributed in group I. The remaining six *Cr*RLK1L members were distributed in groups III and V. Group III contained FER-like proteins identified in ferns, gymnosperms and *A. trichopoda*. Group II consists of four FER-like proteins in lycophytes. Group IV is composed of FER-like proteins in monocots. AtFER and FER-like proteins identified in eudicots constitute the largest group (VI), which is divided into six subgroups (VIa-VIf). The FERs in monocots and dicots were distributed in groups IV and VI, respectively (Fig. 2), suggesting that *FER* genes shared a common ancestor before the divergence between monocots and dicots but underwent some differentiation during evolution.

Among the identified FER proteins, those from eudicots, especially four FER homologs in *Brassica napus*, are closest to AtFER. In addition to FERs in lycophytes, all FER proteins identified from early diverging lineages of land plants (moss and fern), gymnosperms and *A. trichopoda* were clustered with non-FER Arabidopsis *Cr*RLK1L members. The kinship of these proteins is closer to that of 16 non-FER Arabidopsis *Cr*RLK1L members than to that of FER. These results indicate that *Cr*RLK1L family proteins do exist, but no FER homolog seems to exist in mosses, ferns, gymnosperms or *A. trichopoda* in a strict sense. *A. trichopoda* is widely accepted as the earliest differentiated branch of angiosperms and has no obvious genetic relationship with almost all the other angiosperms. These findings suggest that FER originates from the early stages of angiosperm differentiation.

**Fig. 3:**
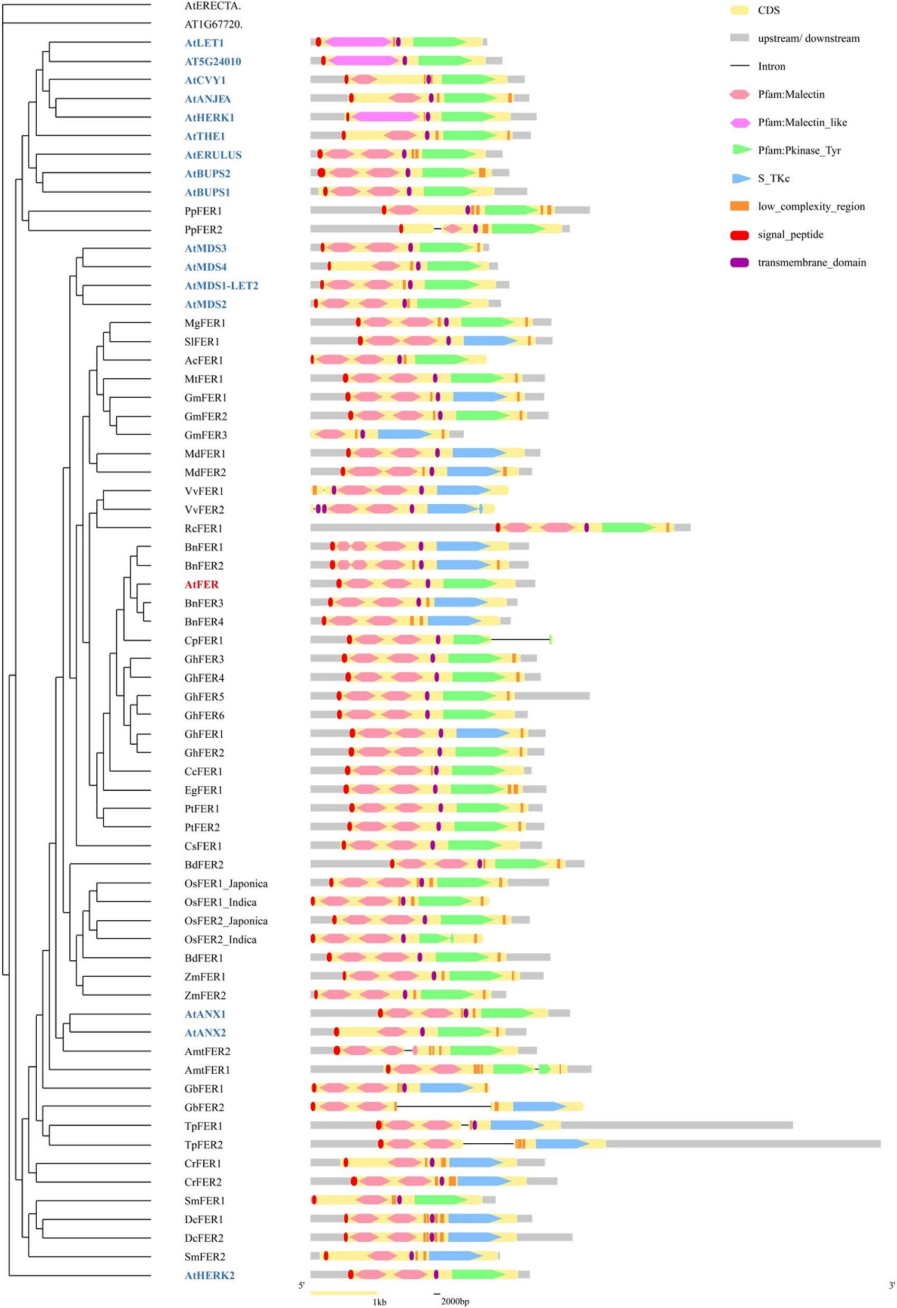
Exon/intron structure and domain organization of the 17 Arabidopsis *CrRLK1L* family members and 50 *FER-like* sequences identified from 26 plant species. The diagram is drawn to scale. All identified FER-like genes are named in the format of "abbreviation of scientific name + FER". Abbreviations of the species: At, *Arabidopsis thaliana*; Pp, *Physcomitrella patens*; Mg, *Mimulus guttatus*; Sl, *Solanum lycopersicum*; Ac, *Aquilegia coerulea*; Mt, *Medicago truncatula*; Gm, *Glycine max*; Md, *Malus domestica*; Vv, V*itis vinifera*; Rc, *Ricinus communis*; Bn, *Brassica napus*; Cp, *Carica papaya*; Gh, *Gossypium hirsutum*; Cc, *Citrus clementina*; Eg, *Eucalyptus grandis*; Pt, *Populus trichocarpa*; Cs, *Cucumis sativus*; Bd, *Brachypodium distachyon*; Os, *Oryza sativa*; Zm, *Zea mays*; Amt, *Amborella trichopoda*; Gb, *Ginkgo biloba*; Tp, *Thuja plicata*; Cr, *Ceratopteris richardii*; Sm, *Selaginella moellendorffii*; Dc, *Diphasiastrum complanatum*

To understand the evolution of *FER* in terms of gene structure, the exons and introns of these 67 genes were analyzed via the online website GSDS. All except 12 *FER* genes contained no introns. The number and phase position of introns differed across the 12 genes carrying introns (Fig. 3). Only *VvFER2* contained two introns, whereas the other 11 introns (*PpFER2*, *VvFER1*, *BnFER1*, *BnFER2*, *CpFER1*, *OsFER2* Indica, *AmtFER1*, *GbFER2*, *TpFER1* and *TpFER2*) each had one. On the basis of the prediction results of domain organization via the online website SMART, overall, the domain compositions of AtFER and its homologous proteins were highly conserved (Fig. 3). All of them were predicted to contain 1 ~ 2 MLDs, a transmembrane domain (except for BnFER4, AmtFER1, AmtFER2, GbFER2 and TpFER2, which bore low-complexity regions in the corresponding position), and an intracellular kinase domain. Nevertheless, variants exist in the malectin domain of FER-like proteins. AtFER and all FER-like proteins except one (GmFER) from seed plants carry two typical malectin domains. However, eight non-FER Arabidopsis *Cr*RLK1Ls and five FER-like proteins (PpFER1, PpFER2, SmFER1, SmFER2 and CrFER1) identified in early diverging lineages of land plants (moss, lycophyte, and fern) had only one typical malectin domain or a large malectin-like domain with the size of two typical malectin domains. This evidence suggests that the FER-like proteins identified in mosses, lycophytes and ferns might have greater homology with some non-FER Arabidopsis *Cr*RLK1L proteins than with FER, which is consistent with the conclusion drawn above that FER originates in the early stage of angiosperm differentiation. In addition, the intracellular kinases were serine/threonine protein kinases in 21 proteins and tyrosine protein kinases in the others (Fig. 3). There seems to be no strong correlation between the type of kinase in FERs and the lineage. The serine/threonine protein kinase domain is present in FER-like proteins of fern, gymnosperm and flowering dicot plant species such as apple, grape and rapeseed, whereas the tyrosine protein kinase domain is distributed in FER-like proteins of moss, *A. trichopoda* and flowering monocot and dicot plant species as well as non-FER Arabidopsis *Cr*RLK1Ls. Intriguingly, some species, such as soybean, cotton and the lycophyte *Selaginella moellendorffii*, contain both types of kinase domains. The functions of these kinases should be verified. Collectively, these results indicate that the *FER* genes were highly conserved during evolution.

## RALF-FER signaling in reproduction, development, and abiotic stress

Certain processes, such as pathogenesis and plant growth and development, are highly similar at the signal transduction level. For example, both the tip growth of fungal hyphae and PTs require cell‒cell communication and penetration into another cell. FER is involved in both processes [51]. To compare the mechanisms underlying RALF-FER-mediated immune signaling and plant growth and development, in this section, we briefly discuss the roles of FER in reproduction, development, and abiotic stress. Readers are also referred to several excellent reviews on multiple functions of FER and the underlying mechanisms [18–26].

### Reproduction

In flowering plants, signaling between the male PT and the synergid cells of the female gametophyte is required for fertilization. RALF-FER has a dual role in ensuring sperm delivery and polyspermy blockade. In *Arabidopsis* mutant *fer*, fertilization is impaired, and its PT fails to arrest and thus continues to grow inside the female gametophyte [13]. FER controls the production of high levels of ROS at the entrance to the female gametophyte to induce PT rupture and sperm release, and this process is Ca^2+^-dependent [52]. PT reception involves three synergid-expressed proteins in Arabidopsis: FER, the glycosylphosphatidylinositol-anchored (GPI-AP) protein LORELEI (LRE), and NORTIA (NTA). When the signal RALFs in the PT are sensed by FER on synergid cells, FER-LRE recruits intracellular NTA to the cell membrane, forming an active Ca^2+^ channel complex that mediates the entry of extracellular Ca^2+^ into synergid cells, thereby inducing PT rupture and sperm release [53]. Recent studies have shown that RALF-FER pairs are involved in the pollen mentor effect. FER/CURVY1/ANJEA (ANJ)/HERCULES RECEPTOR KINASE 1 (HERK1) and the cell wall proteins LRX3/4/5 interact on papilla cell surfaces with autocrine stigmatic RALF1/22/23/33 peptide ligands (sRALFs) to establish a lock that blocks the penetration of undesired PTs. Compatible pollen-derived RALF10/11/12/13/25/26/30 peptides (pRALFs) act as keys, outcompeting sRALFs and enabling PT penetration. pRALFs compete for receptor binding to open the stigmatic lock. The synthetic pRALF peptide successfully triggered wider outcrossing [54]. In stigma papillae, FER forms a complex with ANJ, another member of the *Cr*RLK1L family, to sense autocrine RALF23/33 and activate ROS production through the Rho of Plants 2 (ROP2)-Respiratory Burst Oxidase Homolog D (RBOHD) pathway, which suppresses pollen hydration [55]. The pollen-borne cysteine-rich peptide POLLEN COAT PROTEIN B (PCP-B) competes with RALF33 for binding to FER/ANJ, which results in the suppression of RALF33-induced ROS production in the stigma, facilitating pollen hydration [55]. After successful sperm delivery, FER inhibits the entry of supernumerary PTs into the female gametophyte [56]. FER prevents multiple PTs from entering the same ovule by regulating the accumulation of de-esterified pectin and nitric oxide induced by the first PT in the filiform apparatus [56]. FER, ANJ, and HERK1 receptor-like kinases located at the septum interact with PT-specific RALF6, 7, 16, 36, and 37 peptide ligands to establish polytubey blocks. RALFs secreted from the PT activate FER, ANJ, and HERK1 signaling in septum epidermal cells to establish a polytubey block that prevents the emergence of additional PTs. After successful recognition by the same receptor complex (FER-ANJ-HERK1) in synergid cells, the PT ruptures to release two sperm cells, and fertilization is completed. The polytubey block is then removed as RALFs quickly disappear from the ruptured PT [44].

### Development

FER participates in regulating plant root growth. Compared with wild-type Col-0, the loss-of-function mutant of FER (*fer-4*) presents developmental defects in RH and trichomes [57]. FER, together with LORELEI-like GPI-anchored protein 1 (LLG1), Guanine Exchange Factors 1 (ROPGEF1) and Rac-like GTPases/ROP2 (RAC/ROP2), mediates NADPH oxidase-dependent ROS production to regulate RH polar growthm [57, 58]. In RH, FER also phosphorylates eukaryotic translation initiation factor (eIF4E1), which increases mRNA affinity and modulates mRNA translation, regulating RH protein synthesis to promote RH growth. High levels of ROOT HAIR DEFECTIVE 6-LIKE 4 (RSL4) can exert negative feedback on *RALF1* expression via direct binding to its gene promoter, slowing RH growth and ultimately determining RH cell size [59].

Cell elongation relies mainly on cell wall expansion and central vacuole expansion, which are regulated by FER. The perception of the ligand RALF1 by FER causes the phosphorylation of PM H^+^-adenosine triphosphatase 2 (AHA2) at Ser^899^, further preventing proton transport to alkalize the cell wall [7]. Alkalinization inhibits cell wall expansion through cell wall proteins, such as expansin [60]. The interaction between FER and extracellular LEUCINE-RICH REPEAT EXTENSION 3/4/5 (LRX3/4/5) allows FER to control intracellular vacuolar expansion [61].

### Abiotic stresses

Plants are often confronted with abiotic stresses, such as salinity, high or low temperatures, drought, and heavy metals [46, 62–64]. FER plays an indispensable role in modulating the response to abiotic stress. FER, LRX3/4/5, and RALF22/23 function synergistically in response to high salt stress [46, 65]. High salinity facilitates the cleavage of the RALF22 propeptide by S1P to release mature RALF22 peptides. Mature RALF22/23 interact with FER and thus cause its internalization [46]. The LRX3/4/5-RALF22/23-FER module also controls plant salt stress responses by negatively regulating the levels of jasmonic acid (JA), salicylic acid (SA), abscisic acid (ABA) and ROS accumulation [65]. FER regulates salt tolerance by phosphorylating phytochrome B (phyB). The phosphorylation of phyB regulates dark-triggered photobody dissociation and phyB protein abundance in the nucleus. Salt stress can inhibit the kinase activity of FER, leading to increased phyB protein abundance in the nucleus, which suppresses plant growth and promotes responses to salt stress [66].

FER is involved in the response to temperature stresses [64, 67–70]. Loss-of-function mutants of FER are hypersensitive to both cold and heat stress [67]. At high temperature, a G41S substitution in the extracellular domain of FER caused increased protein turnover and a defect in RH growth [68]. At low temperature, FER interacts with and activates downstream components of TORC to perceive limited nutrient availability and trigger RH growth [69]. The expression of the apple (*Malus domestica*) FER receptor-like kinase gene *MdMRLK2* is rapidly induced by cold. The overexpression of *MdMRLK2* enhances cold resistance, which is attributed to the accumulation of water-insoluble pectin, lignin, cellulose, and hemicellulose [71, 72]. The interaction between MdMRLK2 and the transcription factor MdMYBPA1 leads to increased anthocyanin biosynthesis, particularly under cold conditions [71].

Notably, *Md*MRLK2 is also significantly induced by drought [72]. The overexpression of *MdMRLK2* increases energy levels, activates caspase activity, and results in the accumulation of more free amino acids under drought conditions, indicating that *Md*MRLK2 may be involved in regulating apple drought tolerance by modulating energy metabolism and free amino acid production [72, 73].

FER is involved in metal ion stress. Cadmium (Cd) stress inhibits the expression of *FER* in Arabidopsis roots, and the Cd concentration in *fer-4* mutant roots is reduced to approximately half that in wild-type seedlings. The Cd-induced expression of several genes related to iron (Fe) uptake is downregulated in *fer* mutants [74].

### Putative mechanisms

#### Building the cell surface signal complex

An FER signaling module on the cell surface consists of three core components: FER, a GPI-AP of the LLG family, and RALFs. The *LORELEI* gene is expressed in the synergid cells of the ovule prior to fertilization and encodes LRE, a small plant-specific GPI-AP protein. Lorelei mutants are impaired in terms of female fertility, PT-ovule interactions and sperm cell release, similar to *fer* mutants [75, 76]. LLG1 is the closest homolog of LRE on the basis of amino acid sequences [58]. LRE and LLG1 work individually with FER to regulate different aspects of plant development and reproductive processes in complementary ways. LRE and LLG1 can bind to the extracellular juxtamembrane region on the N-terminal side of the TMD of FER, where they act as coreceptors of the RALF1 peptide. Their interaction can deliver FER from the endoplasmic reticulum to the membrane, where it is required to sense signals and regulate plant growth, suggesting that FER depends on LRE or LLG1 for efficient localization to the cell membrane [58, 76]. X-ray crystallography has revealed the structural basis of RALF binding to its LRE/LLG-FER receptor complex [35]. LLG1 or LLG2 can directly bind to the conserved N-terminal region of RALF23 on the membrane and then form a new action surface to bind to FER under the induction of the ligand RALF23, enabling FER-LLG heterocomplexes to jointly recognize the ligand RALF23. The conserved YISY motif of RALF23 extensively interacts with the loops of LLG2 via a combination of hydrophobic and polar contacts [35].

#### Cross-talk with phytohormone signaling

FER displays exquisite versatility in mediating responses to multiple phytohormones and stresses. FER is an upstream regulator of the RAC/RP signaling pathway, which is activated by auxin and subsequently mediates the derepression of auxin-responsive genes via 26S proteasome-mediated repressor proteolysis [77, 78]. FER also acts as a regulator of auxin to regulate RH development [57]. The *fer-4* mutant presented an increased number of lateral root (LR) branches and delayed gravitropic responses, both of which are caused by defective polar auxin transport. FER controls the polar localization of the polar auxin transporter PIN2 and ultimately contributes to PIN2-associated LR development and gravity responses [79]. FER also mediates root nutating growth through PIN2- and auxin resistance 1 (AUX1)-mediated auxin transport [80].

FER is involved in brassinosteroid (BR) responses. This gene is transcriptionally induced by BRs and is downregulated in the loss-of-function BR mutant *bri1* but upregulated in the constitutive BR-response mutant *bes1-D* [81]. Deslauriers and Larsen [82] reported that the *fer-2* mutation (complete loss-of-function of *FER*) could result in severe hypersensitivity to 24-epibrassinolide (EBL, a hormone of the BR family) in the light and was partially BR insensitive with respect to the promotion of hypocotyl elongation. These results indicate that FER is required for BR responses in light-grown hypocotyls. Notably, the hypocotyl shortening observed in *fer-2*-etiolated seedlings is strongly dependent on ethylene, indicating that FER may regulate cell elongation and hypocotyl growth by balancing the BR and ethylene responses [82]. BZR1, the critical regulator of BR responses, can bind to the promoter of FER to induce its expression in tomatoes, thus regulating downstream BR signaling, which is important for the induction of RBOH1 transcripts, the accumulation of apoplastic H_2_O_2_ and plant heat tolerance [64].

Many FER-dependent pathways are thought to be connected to ABA signaling [67, 83–85]. Recent studies have demonstrated that FER inhibits ABA signaling by increasing the activity of ABA INSENSITIVE 2 (ABI2) phosphatase (a negative regulator of ABA signaling). The interaction between FER and the guanine exchange factors GEF1/4/10 activates the GTPase ROP11/ARAC10, which then physically interacts with the ABI2 phosphatase and enhances its activity [85]. On the other hand, ABI2 and other ABI2-like phosphatases can directly interact with and dephosphorylate FER, leading to the inhibition of FER activity, whereas stress-induced ABA enhances FER signaling through the PYRABACTIN RESISTANCE (PYR)/PYR1-LIKE (PYL)/REGULATORY COMPONENTS OF ABA RECEPTORS (RCAR)–A-type protein phosphatase type 2C (PP2CA) modules [67].

#### Alkalinizing the apoplast

RALF-FER regulates extracellular alkalinization by inhibiting H^+^ transport. For example, RALF1 treatment promotes the phosphorylation of AHA2 by FER [7], leading to the inhibition of H^+^-ATPase activity, moderate alkalinization, and growth suppression. RALF1 exerts biphasic effects on root growth inhibition: a short-term response that inhibits primary root growth through apoplast alkalinization within less than one minute and a long-term (> 1 h) response that involves cross-talk with auxin biosynthesis. The former response is independent of the auxin signaling pathway but is mediated by FER, whereas the latter response is auxin-dependent but FER-independent [86].

#### Sensing cell wall integrity (CWI)

The ECD of *Cr*RLK1L contains two MLDs that share homology with a carbohydrate-binding domain. Plant cell walls are rich in hemicellulose, pectin and other complex carbohydrates. *Cr*RLK1L is assumed to sense and monitor cell wall integrity (CWI). The ECD of FER can indeed physically interact with pectin to protect cell walls from salinity-induced damage [87]. FER can also regulate the formation of leaf epidermis cell shape by sensing cell wall pectin polymers to activate the ROP6 GTPase signaling pathway [58, 88]. In addition, FER regulates sexual reproduction by regulating ovular pectin levels and ovular pectin-induced nitric oxide production when PTs arrive [56].

#### Inducing Ca^2+^ transients

During interactions between the PT and two synergids in flowering plants, FER is necessary to induce a transient Ca^2+^ spike in synergid cells by recruiting the calmodulin-gated calcium channel NTA to the PM [53, 89]. FER is also involved in the increase in cytosolic Ca^2+^ in root cells upon mechanical stimulation [89, 90]. Under salt stress, FER also induces cell-specific Ca^2+^ transients in root cells via an unknown Ca^2+^ channel. These Ca^2+^ transients maintain the CWI of root cells [87].

#### Accumulating ROS

FER positively regulates NADPH oxidase-dependent and auxin-regulated ROS accumulation to promote RH development through the RAC/ROP signaling pathway [57]. In addition, FER-RAC/ROP signaling is involved in the regulation of self-incompatibility, which is important in preventing inbreeding and generating hybrid vigor by promoting NADPH oxidase-dependent ROS accumulation [91]. In contrast, FER can negatively regulate the production of ROS through ABI2, thus inhibiting ABA responses [85]. ROS production is required for salt stress-induced signaling activation, whereas excessive ROS also result in the oxidization and damage of proteins and nucleotides [92]. The Arabidopsis G protein β subunit, AGB1, physically interacts with FER, synergistically regulating salt stress responses by promoting the absorption of K^+^ and inhibiting the absorption of Na^+^ through salt-mediated ROS [40]. FER also regulates heat stress tolerance in tomato via the FER-dependent ROS signaling pathway [64].

#### Recycling via internalization

Some RLKs undergo internalization through endocytosis when they sense ligands. Salt stress causes S1P protease-dependent release of mature RALF22 peptides, inducing the internalization of FER via an endosomal pathway [46].

####  Reprogramming the transcriptome

RALF1-FER signaling enhances the phosphorylation of ERBB-3 BINDING PROTEIN 1 (EBP1) and promotes its nuclear accumulation in plants [93]. EBP1 directly binds to the CALMODULIN-LIKE 38 (CML38) promoter and suppresses its transcription in response to RALF1 [93, 94]. FER can also regulate gene expression via TFs. For example, FER interacts with, phosphorylates, and destabilizes ABA INSENSITIVE5 (ABI5), an important transcription factor in ABA signaling, to mediate cotyledon greening. FER phosphorylates ABI5 at Ser-145 [84]. FER also interacts with, phosphorylates, and stabilizes the basic helix-loop-helix (bHLH) TF PHYTOCHROME-INTERACTING FACTOR 3 (PIF3) to drive root penetration into the soil. PIF3 binds to the promoter of the mechanosensitive ion channel *PIEZO* in the root cap. FER phosphorylates PIF3 at Ser-48, Ser-115, Ser-160, Ser- 196, Ser-201, Thr-234, Thr-335, and Thr-499 [41]. FER can positively control flowering time in Arabidopsis by modulating alternative mRNA splicing and transcript accumulation in FLOWERING LOCUS C (FLC) and MADS AFFECTING FLOWERING (MAF; [95]).

## RALF-FER signaling in plant‒pathogen interactions

The ability of plants to sense and respond to microbial infection determines the outcome of plant–pathogen interactions. As a key component of PRRs, FER is recognized as a core regulator of plant–pathogen interactions. In the following, we revisit the resistance phenotypes of *fer* mutants and discuss the essential contributions of the receptor FER or its homologs and ligand RALFs to plant‒pathogen interactions and their mechanisms.

### Fungal pathogens

Phytopathogenic fungi cause devastating yield losses worldwide and contaminate food with harmful mycotoxins. FER and its homologs in crops or fruits have been implicated in plant immunity to fungal pathogens. In Arabidopsis, FER mutations confer increased resistance to the pathogens *Fusarium oxysporum *[3]*, Golovinomyces* (syn. *Erysiphe*) *orontii *[51], and *Botrytis cinerea* [96, 97] but also increase susceptibility to *Hyaloperonospora arabidopsidis* and *Colletotrichum higginsianum* [51] and *Sclerotinia sclerotiorum* [36, 98, 99]. The fungal biomass after inoculation with *F. oxysporum* was dramatically lower in the *fer-4* plants than in the wild-type plants. In parallel, most of the wild-type plants died, with symptoms characteristic of Fusarium infection, but these symptoms were largely absent in the mutant. Fungal hyphae growing in the *fer-4* mutant underwent cell death. The transcript levels of immune response marker genes are constitutively upregulated in the mutant [3]. In contrast to *F. oxysporum,* inoculation with *S. sclerotiorum* caused significantly larger lesions in *fer‐4* mutants than in wild-type plants, whereas those in FER‐overexpressing lines were smaller than those in wild-type plants. Infiltration with the synthesized peptide RALF22 induced resistance to *S. sclerotiorum* in the wild-type strain, whereas this induction was abolished in the mutant strain [36].

In rice, members of the *FERONIA-like receptor* (*FLR*) gene family trigger a response to the pathogen *Magnaporthe oryzae*, which causes devastating disease. The lesions of the *flr2* and *flr11* mutants were significantly shorter and smaller than those of the wild-type *Hwayoung* (HY) and *Nipponbare* plants, respectively [50]. In the greenhouse, *flr2* resulted in the development of smaller and shorter lesions at the wound-inoculated sites than HY. The fungal biomass of *flr2* was reduced to 10–20% of that of HY. In contrast, *FLR2-OE* plants presented longer lesions and greater fungal biomass than HY plants did. The number of invasive hyphae in the *flr2* plants was significantly lower than that in the HY plants and *FLR2-OE* lines. In the field, the *flr2* mutant was more resistant to rice blast, whereas the FLR2-overexpressing plants presented highly susceptible phenotypes. Compared with the HY plants, the *flr2* plants were more resistant to many blast isolates or physiological races, whereas the *FLR2-OE* lines were more susceptible to these strains than the HY plants were. In summary, mutations in the *FLR2* gene confer a certain degree of broad-spectrum rice resistance. Unexpectedly, this resistance capacity did not cause a growth penalty [50].

In soybean, *Glycine max* LESION MIMIC MUTANT 1 (GmLMM1), a malectin-like receptor kinase structurally close to Arabidopsis FER, negatively regulates resistance to the oomycete *Phytophthora sojae.* Two *GmLMM1* mutant lines presented greater resistance (indicated by lesions and parasite biomass) to *P. sojae* than did wild-type Williams 82 [100].

In tomato, an FER homolog, *Solanum lycopersicum FERONIA Like* (*SlFERL*), positively regulates immune responses to *B. cinerea* invasion. The lesions on the loss-of-function *Slferl* mutant lines following inoculation with *B. cinerea* conidia were significantly larger than those on the wild-type (*Solanum lycopersicum* cv. Ailsa Craig) seedlings [101]. Notably, the resistance of the *Slferl* mutant to *B. cinerea* is distinct from that of the Arabidopsis *fer-4* mutant [96, 97]. The reasons for this discrepancy are unclear. This may be due to the variations in the mutation sites or the pathogen strains or the specific procedures for conidia inoculation and further infection in different studies or to different responses of the FER mutants to pathogens with different lifestyles.

In apple, the expression of *MdMRLK2*, a *FERONIA receptor-like kinase*, was rapidly and strongly induced in the leaves and twigs of *Malus mellana* in response to *Valsa mali* infection. After inoculation with *V. mali*, the lesion areas on both leaves and twigs were clearly larger in the MdMRLK2-overexpressing lines than in the wild-type plants. Compared with wild-type calli, those of the *MdMRLK2* RNAi lines presented significantly smaller lesions and less severe cell death. Therefore, *MdMRLK2* negatively regulates apple resistance to *V. mali *[49].

RALF peptides, such as RALF23 and RALF33, may act as negative regulators of the plant immune response, inhibiting the formation of the signal receptor complex for immune activation [32]. Strawberry (Fragaria × ananassa) rapid alkalinization factor-33-like (FaRALF-33-like) plays a key role in resistance to *Colletotrichum acutatum*, *Botrytis cinerea*, or *Penicillium expansum*. The silencing of FaRALF-33-like expression in *C. acutatum*-inoculated red fruits led to a delay in fruit colonization by the fungal pathogen, and infected tissues presented fewer penetrated infective hyphae than did wild-type fruits. In contrast, *C. acutatum*-inoculated white unripe fruits overexpressing the *FaRALF-33-like* gene presented decreased ontogenic resistance, leading to the appearance of disease symptoms and penetration of subcuticular hyphae, which are normally absent in white unripe fruits. Silencing or overexpressing the *FaRALF-33-like* gene led to different patterns of expression of plant defense genes in strawberry fruits. The *FaRALF-33-like* gene plays an important role in the susceptibility of fruits to the fungal pathogen *C. acutatum *[102]. Future work needs to further determine whether FaRALF-33-like mediates strawberry immunity via its FER homologs in this process.

Fungi produce effectors that allow them to elude or manipulate host defenses. Many plant pathogenic fungi harbor RALF-like peptide genes in their genomes, and fungal RALF mimics have been identified in a wide range of plant pathogens [3, 8], at least some of which are functionally active [8]. For example, the synthesized RALF peptides of the tomato pathogen *Fusarium oxysporum* f. sp. *lycopersici* could inhibit the growth of tomato seedlings and elicit responses in tomato and *Nicotiana benthamiana*, such as ROS bursts, induced alkalinization and MAPK activation. Additionally, this RALF gene is expressed during infection in tomato [8]. Emerging evidence has shown that at least a subset of RALF peptides can be perceived by plant cells. By targeting FER, RALFs secreted by fungi increase their infectious potential and suppress host immunity. The FER of plants senses F-RALF secreted by *F. oxysporum,* increasing the pH around the root and promoting fungal infection [3].

### Bacterial pathogens

Pathogenic plant bacteria are responsible for several crop diseases, threatening the sustainability of agricultural commodities in tropical and temperate regions worldwide [103]. In Arabidopsis, FER positively regulates immunity against *Pseudomonas syringae* pv. tomato DC3000 (*Pst* DC3000) in leaves [32, 104] and roots, especially in the transition zone and elongation zone [47]. FER regulates immune responses to *Pst* DC3000 in a developmental stage-dependent manner. For example, FER mutant seedlings are resistant to *Pst* DC3000 [105], whereas 4- to 5-week-old FER mutants are hypersensitive to this pathogen [32, 104]. In addition, mutations in FER increase resistance to the invasion of *Ralstonia solanacearum* GMI1000, a soil-borne bacterial pathogen [106, 107]. The exogenous application of chemical inhibitors that specifically inhibit the kinase activity of FER or its homologs from different plant species enhances plant root immunity to *R. solanacearum* [106]. These results indicated that FER negatively regulates immunity in roots against *R. solanacearum*.

Microbial invasion causes changes in the composition and abundance of the plant microbiota, which consequently affects plant immunity. FER-mediated immunity in roots could shape the rhizosphere microbiome. Mutation of FER assembles a rhizosphere microbiome that is beneficial for alleviating inorganic phosphate starvation. The beneficial microbiome includes *Pto* DC3000 and the genera *Flavobacterium*, *Pseudomonas* and *Delftia* [4]. The loss-of-function mutants *fer-4* and *fer-8* displayed rhizosphere pseudomonad (*Pseudomonas fluorescens*) overgrowth. *fer-8* had a rhizosphere microbiome enriched with *Pseudomonas fluorescens* without phylum-level dysbiosis. Eight of the nine tested Pseudomonas strains were enriched by two- to 18-fold in the rhizosphere of *fer-8* relative to that of the wild-type plants. The *fer-8* microbiome is beneficial and can promote the growth of the next generation of plants [97].

In rice, FERONIA-like RLK 7 (*FLR7*) negatively regulates rhizosphere oxygen levels in roots to shape the rhizosphere microbiome, especially the dominant anaerobic-dependent genus *Anaeromyxobacter*. A representative *Anaeromyxobacter* strain improved submergence tolerance in rice via FLR7 [108].

In soybean, plant defense-related genes were highly enriched in the *Gmlmm1* mutant plants. Both lines presented greatly increased resistance to *Pseudomonas syringae* pv. *glycinea*, and *P. syringae* pv. *phaseolicola*, which can cause halo blight in beans [100].

### Nematodes

Root-knot nematodes (RKNs) are among the primary instigators of worldwide crop damage. Two RALF-like genes, *MiRALF1* and *MiRALF3*, from *Meloidogyne incognita* are expressed in the esophageal gland and are highly expressed during the parasitic stages of nematode development. They possess the typical activities of plant RALFs and can directly bind to the ECDs of FER to modulate specific steps of nematode parasitism-related immune responses and cell expansion. Both MiRALF1/3 and FER are required for RKN parasitism in both Arabidopsis and rice. FER and its rice homolog FLR1 have similar functions in response to *M. incognita* [5]. In addition, *GmLMM1* mutants exhibit increased resistance to the RKN *M. incognita* in soybean [17].

### Putative mechanisms

**Fig. 4:**
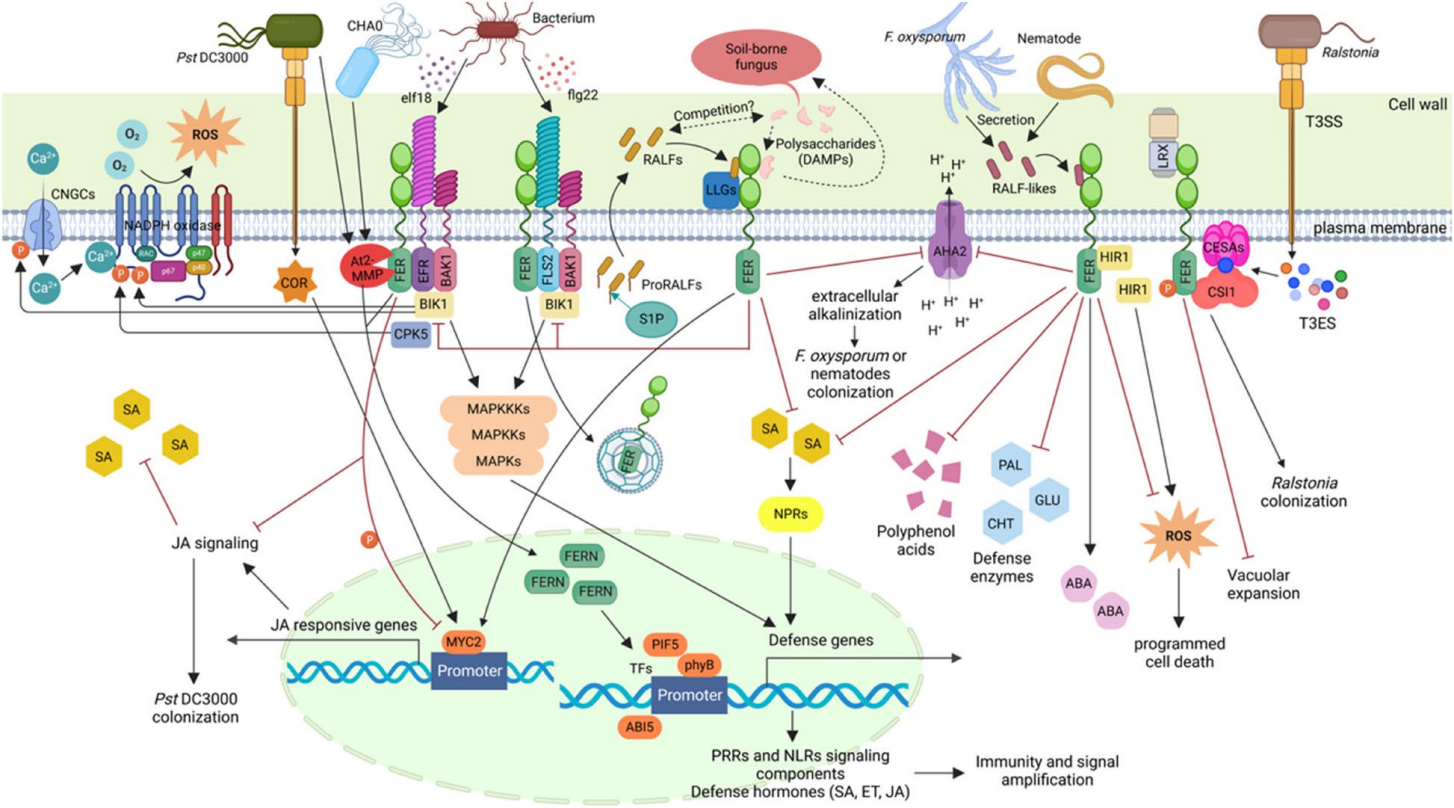
Complex roles of FER in plant immunity. FER serves as a scaffold to promote the formation of FLS2/EFR-BAK1 complexes triggered by flg22/elf18, activating downstream PTI reactions, including Ca^2+^ oscillations, NADPH oxidase-induced ROS bursts, and MAPK cascade reactions. FER and its coreceptor LLGs interact with RALF23, which is cleaved by S1P, to form RALF-FER-LLGs, which inhibits the formation of FLS2/EFR-BAK1 complexes. Moreover, flg22 stimulation significantly promotes the lateral mobility and dissociation of FER from the plasma membrane, as well as the endocytosis and recycling of FER. F. oxysporum and nematodes can secrete RALF analogs that bind to FER, thus inhibiting H + efflux mediated by AHA2 and regulating related immune responses, ultimately promoting infection. Pst DC3000 secretes COR to utilize MYC2 in the JA signaling pathway, inhibiting SA accumulation and promoting infection. FER phosphorylates MYC2 to reduce its stability. After the perception of RALF23, FER stabilizes MYC2 again and activates JA signaling, negatively regulating plant immunity. FER senses CWI through interaction with LRX or CSI1 to regulate vacuole expansion or plant resistance to R. solanacearum . Rhizobacterium CHA0 and Pst DC3000 facilitate the accumulation of the metalloproteinase At2-MMP, which spatially cleaves FER. The truncated form of FER (FERN) is translocated into the nucleus and activates MAMP responses via the promotion of TFs to increase defense gene expression. The apple FER receptor-like kinase MdMRLK2 increases ABA levels but reduces SA levels and inhibits polyphenol acid accumulation, defense enzyme activities and HIR1-mediated HR, negatively regulating resistance to Valsa canker. Refer to the text for details. Positive regulatory actions are indicated by arrows, whereas negative regulatory actions are indicated by inhibitors. The solid arrows and inhibitors are supported by the literature, whereas the dashed arrows and inhibitors are hypothetical. The gradient arrows indicate the movement directions of Ca^2+^, H^+^, COR and the effectors

The RALF-FER signaling pathway is tightly regulated, as excessive activation of FER leads to autoimmunity and growth inhibition [100, 105]. The abovementioned studies highlight the diverse mechanisms that FER might employ to modulate plant immune processes. How RALF-FER-dependent pathways are differentially activated and coordinated in different immune processes remains an interesting question to address. Here, we discuss several mechanisms underlying the FER-mediated plant immune response (Fig. 4). Notably, these mechanisms are not completely independent but overlap during one type of pathogenesis, and most of them could also underlie FER-mediated growth, development, reproduction and abiotic stresses.

#### Building the PRR complex

FER facilitates the ligand-induced complex formation of the immune receptor kinases EFR and FLS2 with their coreceptor BAK1 to initiate immune signaling, acting as a scaffold that modulates PRR assembly [32]. Arabidopsis RALF23 can inhibit plant immunity by recruiting FER and inhibiting the formation of this complex [32]. FER-mediated changes in the root microbiota are likely achieved through the regulation of PRR complex formation in roots [4]. Similarly, GmLMM1 interacts with GmFLS2 and GmBAK1 and negatively regulates PTI by suppressing flagellin-induced GmFLS2–GmBAK1 complex formation [100].

#### Regulating the dynamics of FLS2 and BAK1 at the PM

Immune receptor complexes are subject to dynamic regulation to allow tight control of the intensity and duration of plant immune responses. Pathogen infection often induces the rearrangement of host PM constituents and the formation of a membrane microdomain, a compartment distinct from the surrounding membrane in composition, structure, and biological function. The dynamic regulation of PM nanoenvironments is considered a key fundamental aspect of PTI. FER can orchestrate nanodomain organization on the PM, increasing the plant immune response. In addition to acting as a scaffold, FER regulates the PM nanoscale organization of FLS2 and BAK1: FLS2 molecules are more dispersed and dynamic, whereas BAK1 is more structured and static in *fer-4* than in the wild type [109]. The perception of RALF peptides by FER actively modulates PM nanoscale organization to regulate plant immune signaling [109].

#### Alkalinizing the apoplast

All the RALF peptides can induce alkalinization. For example, RALF1 binds to FER and facilitates its phosphorylation and thus inhibits the activity of hydrogen pumps and H^+^ efflux, thereby inducing extracellular alkalization [7]. Although immune inhibition is not a general property of RALFs and is independent of alkalinization activity, FER-mediated extracellular alkalinization helps fungal pathogenesis and nematode parasitism. *F. oxysporum* encodes a functional RALF-like peptide with alkalinizing and growth-regulatory activity toward plants [3]. Like RALF1, nematode RALF-like bacteria rapidly alkalize the extracellular medium, suggesting that nematode RALF-like bacteria may also manipulate cell expansion and induce cell wall modification to facilitate giant cell development. Nematode RALF-like bacteria induce alkalinization in an FER-dependent manner, which may modify plant cell walls and thereby facilitate parasitism [5].

Changes in the extracellular pH caused by pathogen infection can alter the activity of both plant and microbial cell wall-modifying proteins and cell wall-degrading enzymes and modify polysaccharide interactions, enabling restructuring of the cell wall that favors colonization. The changes in the extracellular pH and their influence on plant‒pathogen interactions are time-, organ-, and even cell layer dependent. In the future, the complexity of extracellular pH changes during plant‒pathogen interactions needs to be untangled with greater spatiotemporal resolution in different pathosystems.

#### Sensing CWI

The plant cell wall serves as an exoskeleton that maintains the integrity of each cell, the overall plant architecture, and a barrier to invasive damage, such as physical wounding and pathogen penetration. Monitoring the cell wall status is indispensable for plant life. CWI maintenance may compensate for the immunodeficiency caused by defective PTI signaling. The *fer* mutant displays a burst-cell phenotype [110]. As a candidate cell wall sensor, FER modulates intracellular vacuolar expansion by sensing extracellular matrix features through interactions with extracellular LRX proteins [61]. The LRR domain of LRXs likely interacts with FER, and the EXTENSIN domain binds to cell wall components, thereby sensing and conveying extracellular signals to cells [61]. FER-mediated crosstalk between immunity and CWI sensing is required for FER-regulated PTI signaling [109].

The cell wall consists of cellulose, hemicellulose, pectin, and structural proteins. These components determine CWI. Pectin and pectin-derived products can be derived from cell wall breakdown under pathogen attack, and pectin sensing by FER might integrate CWI maintenance with immune signaling. A lack of FER results in the inability of plants to adjust their pectin composition in response to biotic stress [96]. CELLULOSE SYNTHASE (CESA)-INTERACTIVE PROTEIN 1 (CSI1) regulates the activity of CESA complexes at the PM, determining cellulose biosynthesis. CSI1 could contribute to the specific FER-mediated regulation of immunity through the formation of a CSI1-FER complex. CSI1 could be directly involved in the sensing of alterations in CWI through its association with FER and/or other CWI sensor(s), forming a module that differentially regulates resistance against pathogens in roots and shoots [107].

#### Accumulating ROS

ROS act as toxic molecules to fight invading microbes and as signaling molecules to activate plant defenses. The FER–LLG1 complex binds to ROP-GEF, which activates the Rho-like GTPase RAC/ROPs, leading to the activation of the NADPH oxidase RBOH and a subsequent MAMP-induced ROS burst [55, 57]. In addition, FER can activate ROS generation via the RPM1-induced protein kinase (RIPK)–NADPH oxidase RBOH D (RBOHD) module [36]. FER is required for flg22-, elf18-, and chitin-triggered ROS production [32]. Mutants of FER exhibit a conspicuous defect in the PAMP-triggered accumulation of ROS [96, 97, 105]. Compared with wild-type plants, *FLR2* mutation resulted in much greater amounts of ROS (H_2_O_2_) at the penetration sites of leaf cells, whereas less H_2_O_2_ was produced in the leaf cells of the *FLR2-OE* lines in response to rice blast [50]. The overexpression of the *GmLMM1* gene in *N. benthamiana* severely suppressed flg22-triggered ROS production. Compared with Williams 82, the *Gmlmm1-1* and *Gmlmm1-2* mutants presented significantly increased ROS accumulation [84]. FER-mediated ROS production regulates the levels of beneficial pseudomonads in the rhizosphere microbiome [97]. RALF22 promotes Pep-induced ROS generation [36], whereas RALF23 reduces elf18-induced ROS production [32].

ROS physically block the intruder path by strengthening the cell wall via the induction of callose synthesis genes and spatially restricted lignin deposition, which contributes to CWI. Therefore, FER may maintain the cell wall status via ROS.

#### Activating MAPK signaling

The activation of MAPK cascades is an early signaling event in both PTI and effector­ triggered immunity (ETI), which subsequently regulates immune outputs, including hormone production, callose deposition, and transcriptional reprogramming. MAPK cascades are evolutionarily conserved in plants. The inorganic phosphate starvation-mediated MAPK inhibition observed in the wild type was nearly completely abolished in *fer-4* mutants under LP conditions [4]. In Arabidopsis, MPK3 and MPK6 are highly phosphorylated by RALF22 synthesis [36]. MAPK activity in Col-0 seedlings was promptly activated to different extents upon stimulation with RALF1, *Mh*RALF3, and *Mi*RALF1/3 [5]. SlFERL fine-tunes MAPK signaling: SlFERL interacts with SlMAP3K18, a mitogen-activated protein kinase kinase kinase, to tune SlMAP2K4 kinase activity [101].

#### Recycling via internalization and endocytosis

Protein accumulation of PRR immune components is under tight control and is affected by endocytosis. Upon ligand binding, several RLKs have been shown to undergo internalization through endocytosis [111], which may remove receptor molecules from the membrane pool. The flagellin peptide flg22 induces lateral diffusion, disturbs the initiation of endocytic processes, and increases the retention time of FER on the PM. FER undergoes constitutive endocytosis and recycling and is sorted at the TGN via a clathrin-dependent pathway [112].

#### Being cleaved by a metalloproteinase

A very recent study revealed that cleaved FER positively regulates localized immune activation in the transition and elongation zones of Arabidopsis roots [47]. When the seedlings were inoculated with rhizobacterium CHA0 and DC3000, the root cells facilitated the maturation of RALF23 and the accumulation of the metalloproteinase At2-MMP. At2-MMP spatially cleaved FER at its intracellular juxtamembrane domain. The truncated form of FER is translocated into the nucleus and activates the MAMP response in the transition zone and elongation zone of the root [47].

#### Destabilizing MYC2 via phosphorylation

The transcription factor (TF) MYC2, a master regulator of JA signaling, activates NAC transcription factors, which function to inhibit the accumulation of SA and compromise plant immunity. FER interacts with, phosphorylates, and destabilizes MYC2 [104], which positively regulates plant immunity to the hemi-biotrophic bacterium *Pst* DC3000. RALF23 inhibits FER’s negative regulation of MYC2 stability and promotes JA signaling, favoring bacterial infection [104]. Like Arabidopsis RALF1, *Mi*RALF1 and *Mi*RALF3 reduce the protein level of MYC2 during the parasitism process [5].

#### Reprogramming the host transcriptome

Microbial infection induces transcriptional reprogramming in plants, which provides plants with the flexibility to launch rapid and appropriate defenses. Defense-responsive genes are constitutively upregulated in *fer* mutants. MYC2 functions downstream of FER and at least partially accounts for the positive role of FER in bacterial defense responses [104]. FER also regulates other TFs, such as PIF3 [63], ABI5 [84], and phyB [66]. The evidence that truncated FER can be translocated to the nucleus and that MAMP marker genes present different expression patterns in *fer-4* roots [47] suggests that FER can regulate defense-responsive gene expression via MYC2 or other unknown TF(s).

## Perspective

In the past decade, extensive studies have demonstrated that microbial effectors enhance virulence often by directly interfering with PRR-mediated signaling and that these effectors can also promote infection by mimicking RALF to indirectly interfere with immune signaling through cross-talk between different signaling pathways. Despite the recent increase in FER-related studies, the understanding of the roles and mechanisms of RALF-FER in plant‒pathogen interactions remains to be further strengthened.

First, the specificity of RALFs’ interactions with FER, the competing relationships between RALFs and other peptides, and the dose-dependent effect of RALFs will continue to be a fertile ground for investigation: (1) Although FER senses various RALFs, such as RALF1 [7] and RALF23 [4, 32, 47, 97, 104] from host plants and Fusarium-RALF [3], MiRALF1, and MiRALF3 are excreted from pathogens [5], the binding and functional specificity of RALF ligands to FER and the mechanisms regulating the formation of specific FER receptor complexes remain unclear. Furthermore, RALF23 and RALF22 are closely related [16] but play opposite roles: RALF23 inhibits an elf18‐induced ROS burst and plays a negative role in plant immunity [16, 32], whereas RALF22 directly induces an ROS burst and plays a positive role in plant immunity [36]. Whether their distinct roles in plant immunity are related to the binding and functional specificity of RALF ligands to FER is not well understood. (2) FER also senses other ligands, such as PCP-B [55] and pectin [87, 88, 113]. PCP-Bs from compatible pollen outcompete stigma RALF33 for binding to FER, which disengages FER-to-ROS signaling, reduces stigmatic ROS levels to unlock the gate, and facilitates pollen hydration and germination [55]. De-methylesterified pectin binding to the ECDs of FER stimulates the activation of RAC/ROP, which impacts the microtubule cytoskeleton, a major cellular target system of RAC/ROPs, and underlies the mechanical stress sensing encountered during pavement cell morphogenesis [88, 113]. Whether pathogens hijack FER signaling by competing with RALF binding via PCP-B and/or pectin ligands to improve their virulence is still an open question. (3) Acting as ligands of FER, both RALF22 [36] and RALF23 [47] have dose-dependent effects on plant immunity. Whether this property is common to all members of the RALF family remains an interesting question for future research.

Second, the existence, molecular basis, and mode of action of RALF-triggered FER-independent immunity is still unknown. To date, most studies [3–5, 17, 32, 36, 47, 97, 104] have shown that RALF-triggered immunity is dependent on FER. Intriguingly, a very recent study revealed that RALF22 forms a complex with pectin, acting as a cell wall-structuring component to regulate root hair growth, which is unexpectedly independent of FER [38]. This finding suggests that this feature of RALF22 also exists in RALF-triggered plant immunity. Therefore, whether the cell wall-structuring role of RALF22 [38] contributes to defenses against pathogens will be interesting to test.

Third, FER-dependent CWI sensing and maintenance deserve future study. Plant defense necessitates CWI maintenance, which is sensed and regulated by FER. The cell walls of the *fer-4* mutant are thinner than those of the wild type [114]. The cell wall thickness is related to the resistance or susceptibility of *fer* mutants. Whether FER functions in plant immunity through the sensing of CWI, how CWI disturbance during infection is linked with plant immune responses, and the involvement of FER in plant immunity remain largely unknown. Additionally, a phytocytokine-receptor pair, SCOOP18-MIK2, was recently found to sense CWI [115]. Whether and how FER and SCOOP18-MIK2 synergistically function in sensing CWI to regulate plant immunity await investigation.

Pathogens exploit various infection strategies to evade or counter host immunity and eventually cause disease. Among these strategies, evasion of plant immunity through dampening host recognition or subsequent immune signaling is crucial infection strategies. To avoid microbial evasion of host immunity, a commonly advocated strategy to increase resistance durability is stacking immune receptor genes. FER-related studies have already been extended to major crop species. Mechanistic concepts advanced from studies in model systems will be rich grounds on which to build translational efforts to improve how crop species might cope with environmental challenges. RALF22 directly elicits a variety of typical immune responses and induces resistance against the devastating necrotrophic pathogen *S. sclerotiorum*. RALF22 triggers immune responses and augments Pep3‐induced immune signals in an FER‐dependent manner [36]. How could RALFs be exploited in crop protection? Although FER is considered a core signaling sector in plants, no manipulation of the RALF–FER ligand–receptor pair for improved crop disease resistance has been reported thus far. In the future, this ligand‒receptor pair may be deployed for sustainable crop disease control.

## Data Availability

All data are available in the manuscript.
